# An Unexpected Case of Somatic Mosaicism of the Dutch p16-*Leiden* Founder Variant in the *CDKN2A* Gene

**DOI:** 10.1155/crig/6261903

**Published:** 2025-08-28

**Authors:** M. van der Meulen, J. T. van Wezel, D. Terlouw, J. Morreau, E. M. P. Steeghs, R. van Doorn, M. E. van Leerdam, A. M. Onnekink, M. C. de Ruiter, N. van der Stoep, T. P. Potjer

**Affiliations:** ^1^Department of Clinical Genetics, Leiden University Medical Center, Leiden, the Netherlands; ^2^Department of Pathology, Leiden University Medical Center, Leiden, the Netherlands; ^3^Department of Dermatology, Leiden University Medical Center, Leiden, the Netherlands; ^4^Department of Gastroenterology and Hepatology, Leiden University Medical Center, Leiden, the Netherlands; ^5^Department of Anatomy and Embryology, Leiden University Medical Center, Leiden, the Netherlands

## Abstract

*CDKN2A* is the primary high-risk predisposition gene for familial cutaneous melanoma. In the Netherlands, most carriers of pathogenic germline variants in *CDKN2A* harbor a unique, population-specific founder variant, c.225_243del, commonly referred to as p16-*Leiden*. For decades, this distinctive 19 base-pair deletion in *CDKN2A* had been identified exclusively as a germline variant. Here, we report an exceptional case of somatic mosaicism for the p16-*Leiden* variant in an Irish male with a concurrent diagnosis of Kartagener's syndrome but no history of malignancy. The variant was first identified through targeted next-generation sequencing (NGS) of a fundic gland polyp in the distal esophagus, showing a variant allele frequency (VAF) of 40%. Subsequent analysis also detected the variant in the patient's buccal swab DNA (VAF 0.3%), while it was notably absent in multiple other tissue samples, including blood, urine, skin, and several additional samples from the proximal gastrointestinal tract. We explore several hypotheses that could explain these intriguing findings.

## 1. Introduction

The Cyclin-Dependent Kinase Inhibitor 2A (*CDKN2A*) gene is a key tumor suppressor gene that is frequently involved somatically in tumorigenesis. It encodes two distinct proteins, p16INK4a and p14ARF, that result from the splicing of two distinct exon 1 sequences (1α and 1ß) that are translated in alternate reading frames. These proteins act in separate pathways: the p16-retinoblastoma (Rb)-pathway, which controls cell cycle G1-phase exit, and the p14ARF-p53 pathway, which induces cell cycle arrest or apoptosis [1]. In the context of hereditary cancer, *CDKN2A* is known as the primary high-risk predisposition gene for familial cutaneous melanoma (OMIM #155601), with inactivating germline variants found in approximately 10%–40% of melanoma-prone families [2, 3]. Variant carriers in these families face a substantially elevated lifetime melanoma risk, ranging from 50% to 70%, and frequently develop multiple primary melanomas typically starting at a relatively young age (< 45 years) [4–6]. Variant carriers also have an increased risk for other cancers, especially pancreatic cancer, with an estimated lifetime risk of 15%–20% [7, 8].

In the Netherlands, the vast majority of *CDKN2A* germline pathogenic variant carriers have a specific Dutch founder variant known as p16-*Leiden*, a 19-base-pair deletion in exon 2 (c.225_243del) that causes a frameshift and premature stop codon and results in truncated p16INK4a and p14ARF proteins [9, 10]. Since its discovery in 1995 [11], the p16-*Leiden* founder variant has been identified exclusively as a hereditary germline variant linked to cancer predisposition, primarily in the Dutch population or among those of known Dutch ancestry. However, this report presents an exceptional case of somatic mosaicism for the p16-*Leiden* variant in an individual with no known Dutch ancestry.

## 2. Case Presentation

The patient is a 58-year-old male of Irish descent with a clinical diagnosis of Kartagener's syndrome, based on the presence of situs inversus in combination with characteristic features of primary ciliary dyskinesia (PCD). These include a long-standing history of recurrent airway infections and bronchiectasis, chronic rhinosinusitis, and chronic otitis media leading to mixed hearing loss. His additional medical history includes diaphragmatic hernia, hepatic steatosis, and osteoporosis with multiple fractures. He has no history of malignancies. The eldest of four siblings, he has two younger brothers and one sister. His sister was diagnosed with nasal cancer when she was 48-year-old. His father had lung cancer and died in his 80s, while his mother had a tumor in the leg of an origin unknown to the patient. He has no children.

At the age of 56, the patient underwent a resection of a 15 mm stalked polyp at the z-line in the distal esophagus, which had been an incidental finding on a CT scan. On pathological examination, it was classified as a fundic gland polyp with high-grade dysplasia. Subsequent targeted next-generation sequencing (NGS) of the tumor was performed using an in-house developed panel covering hotspot mutations in genes frequently mutated in cancer. This analysis revealed the characteristic pathogenic variant in *CDKN2A,* c.225_243del, also known as p16-*Leiden*, with a variant allele frequency (VAF) of 40% in the dysplastic region of the polyp and, in a separate analysis, 63% in the nondysplastic region. No other pathogenic variants were detected. To confirm the likely hypothesis of this being a germline variant, molecular analysis was performed in a gastric antrum biopsy showing nondysplastic mucosa, which had been taken 6 years earlier. To our surprise the variant was not detected in this gastric tissue, suggesting either a highly unusual somatic mutation acquired during polyp formation or a potential somatic (embryonal) mosaicism for the p16-*Leiden* variant perhaps not confined or causally related to the esophageal polyp itself. The sequencing quality was sufficient to detect any p16-*Leiden* variant in the gastric tissue, while comparison of SNP profiles confirmed that the recent esophageal and prior antral gastric biopsies belonged to the same person, thereby excluding the possibility of sample replacement.

**Figure 1:**
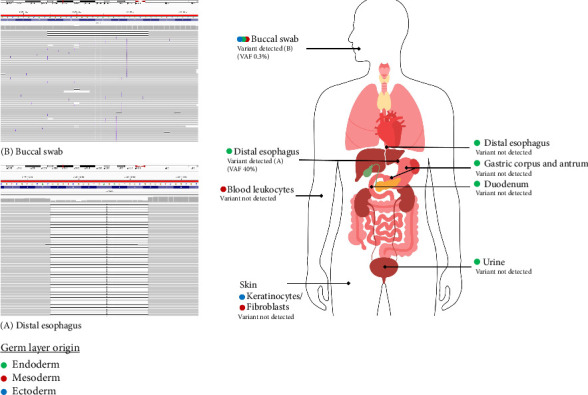
Detection of the p16-*Leiden* variant across different tissues. (A) and (B) show variant detection in the 9p21.3 locus by next-generation sequencing in DNA from the distal esophagus and buccal swab, respectively. The schematic on the right summarizes variant detection status in tissues of endodermal (green), mesodermal (red), and ectodermal (blue) origin. The p16-*Leiden* variant was detected in tissue samples from the distal esophagus (VAF 40%) and buccal swab (VAF 0.3%), but not in other sampled tissues.

In our initial workup for somatic mosaicism of the p16-*Leiden* variant, we examined DNA samples from blood, urine, and a buccal swab. Although the variant was not detected in DNA samples from blood and urine (2800 reads and 985 reads, respectively), in the buccal swab DNA sample the variant was detected at a very low VAF of 0.3% (3/1111 reads, and at repeated analysis 5/1636 reads). Comparison of SNP profiles again confirmed that the buccal swab came from the same person as the esophageal and gastric biopsies. This finding suggested a very low-level somatic mosaicism for the p16-*Leiden* variant and prompted us to further investigate additional tissue samples of the upper gastrointestinal tract and the skin. In two biopsies of the gastric corpus and duodenum taken 6 years earlier, both showing nondysplastic mucosa, the variant was absent (420 reads and 449 reads, respectively). Additional tissue samples of the distal esophagus were acquired, again demonstrating the absence of the variant in both glandular cells and epithelial cells (4451 and 1037 reads, respectively). We then obtained biopsies taken from different areas of the skin. In two of these biopsies, from the skin of the elbow crease and the back—both lacking visible nevi and containing only keratinocytes and fibroblasts without readily discernible melanocytes—the variant was again absent (in 794 and 726 reads, respectively). A further biopsy was taken from a pigmented skin lesion on the left arm, which unfortunately did not contain nevus cell nests, and the variant was once more absent (2783 reads). A fourth biopsy of a nevus on the right upper arm containing melanocytes could not be analyzed due to the low quality of the material. Unlike most patients with hereditary melanoma due to a germline pathogenic *CDKN2A* gene variant, our patient's skin showed very few melanocytic nevi or other pigmented skin lesions, so no additional biopsies of pigmented skin lesions could be attempted. A summary of the sequencing results across various tissue samples is shown in Figure 1.

## 3. Discussion

To the best of our knowledge, this remarkable case represents the first documented instance of *CDKN2A* gene variant mosaicism in the medical literature. Notably, it involves mosaicism of a distinct 19-base-pair deletion in the *CDKN2A* gene, previously observed only as a germline founder variant. Potential technical issues that might explain these unexpected findings were rigorously excluded. Sample replacement was discounted using SNP profiles, and variant miscalling was addressed through extensive and repeated analyses. The presence of this specific variant at very low mosaic levels in an individual of non-Dutch descent remains puzzling, though several hypotheses may be considered.

Firstly, could there be an as yet unidentified connection between the findings in our patient and the p16-*Leiden* variant as a known germline founder variant? The p16-*Leiden* founder variant in melanoma families has been traced back to a common ancestor in the year 1707, with all affected families believed to share a common founder long predating 1700 [12]. There is no evidence that the variant has arisen multiple times as a *de novo* germline variant, which would in fact be highly unlikely in the case of a specific 19-base-pair deletion not located in a known mutational hotspot or fragile site. Additionally, population databases such as the Genome Aggregation Database (gnomAD) show no recurrent variants at this location in the general population [13]. Although the *CDKN2A* gene is frequently involved somatically in melanoma [14, 15], pancreatic cancer [16], and other tumors [17], to our knowledge the specific p16-*Leiden* variant has never been identified as a purely somatic variant in any tumor. Moreover, the *CDKN2A* gene is not typically involved somatically in gastrointestinal polyps, as seen in our patient. These considerations raise the hypothetical possibility of a revertant mosaicism in our patient, a phenomenon observed in several genodermatoses where the loss of a germline variant in one or more cells during embryonic development coincidentally provides those cells with a selective growth advantage, resulting in a somatic mosaicism of the initial germline variant [18]. However, since the p16-*Leiden* variant itself confers a growth advantage, no growth advantage would be expected if this variant was lost during embryogenesis. Without a family history of melanoma or pancreatic cancer, and in the presumed absence of a Dutch ancestry, this hypothesis seems highly unlikely to be relevant in this particular case.

In our patient, the p16-*Leiden* variant therefore most likely emerged *de novo,* either at some point during embryonic development or later in life as a somatic tumor mutation. Detection in buccal swab DNA was remarkable and initially suggested an embryonic origin, likely originating in endodermal cells that later formed the gastrointestinal epithelium given the high frequency of the variant in esophageal polyp tissue. It is unlikely to have arisen earlier, as this would typically result in the variant being present across a much broader range of cell types and tissues. While a buccal swab primarily collects cells of ectodermal origin (from the oral mucosa epithelium of the proximal oral cavity) and mesodermal origin (leukocytes), it may also include (some) cells of endodermal origin that migrated from the distal oral cavity and gastrointestinal tract to the proximal oral cavity at a later stage of embryonic development [19]. The strict boundary between the ectoderm and endoderm, following the disappearance of the oropharyngeal membrane, is nowadays a topic of considerable debate, highlighting the complexity of mouth tissue origins [20, 21]. Although rare, heterotopic gastrointestinal mucosa has been described in the oral cavity, typically presenting as cysts or nodules [22, 23], and could serve as another potential source of cells of endodermal origin in the proximal oral cavity. However, given that our patient had no history of oral cysts or nodules, this also seems an unlikely explanation for the presence of the variant in his buccal swab DNA.

Alternatively, the p16-*Leiden* variant could have arisen as a purely somatic tumor mutation in the esophageal polyp itself, with mutant cells from the polyp subsequently migrating to the oral cavity. The phenomenon of ‘field cancerization' has been hypothesized as the mechanism for, amongst others, some *APC* mosaicisms in the colon and the spread of somatic oncogenic clones in inflammatory bowel disease [24, 25]. In patients with esophageal squamous cell carcinoma or adenocarcinoma, field cancerization has been detected optically (but not genetically) in the oral mucosa [26]. This might suggest that migration of mutant cells from the esophageal polyp to the oral cavity is indeed possible, explaining the variant's presence in the buccal swab DNA. This argument is further bolstered by its absence in all other examined tissue samples (including additional samples from the distal esophagus), which is rather atypical in case of an embryonic mosaic. An additional intriguing, albeit purely theoretical, hypothesis—and the only potential link to the patient's Kartagener's syndrome that we can currently suggest—is that the very low-frequency presence of the p16-*Leiden* variant in the buccal swab DNA is due to contamination by mutant cells originating from the esophageal polyp as a result of gastrointestinal reflux secondary to the patient's diaphragmatic hernia. However, since the buccal swab was taken several months after the polyp's resection, this would imply that the mutant cells persisted in the oral cavity long after the original source was removed.

In view of our unprecedented findings, screening advice for this patient was based on several distinct considerations. A pancreatic biopsy was considered for this patient due to the variant's clinical relevance, as pancreatic cancer surveillance is recommended for germline carriers of the p16-*Leiden* founder variant. However, this was deemed too invasive given the low yield in previous investigations and because a low VAF in pancreatic tissue would likely be insufficient to justify pancreatic surveillance. Given the ectodermal (neural crest) origin of melanocytes, we did not expect the p16-*Leiden* variant to be present in these cells. Furthermore, as the patient had never had a melanoma, did not carry the p16-*Leiden* variant in any of the skin biopsies, and had very few melanocytic nevi, his melanoma risk was considered near population risk. Annual melanoma surveillance by a dermatologist was therefore not considered necessary. The patient was nonetheless advised to regularly inspect his skin and visit the dermatologist in case of any dermatological changes. If further cases of somatic *CDKN2A* variant mosaicism are identified in the future, screening recommendations should be tailored to individual characteristics.

In conclusion, we report an exceptional case of somatic mosaicism for a distinct 19-base-pair deletion in the *CDKN2A* gene, historically recognized solely as a Dutch population-specific germline founder variant in the context of hereditary melanoma and pancreatic cancer. The variant's presence in DNA from both an esophageal polyp and a buccal swab suggests an embryonic origin of the variant in this patient. However, its complete absence in the many other tissue samples examined is atypical, allowing room for an alternative hypothesis in which somatically mutated tumor cells may have migrated from the esophageal polyp to the oral cavity later in life. Whatever its origin, the *de novo* occurrence of this specific *CDKN2A* variant is unprecedented since its earlier emergence as a population-specific founder variant many centuries ago.

## Data Availability

The data that support the findings of this study are available on request from the corresponding author. The data are not publicly available due to privacy or ethical restrictions.

## References

[1] Sherr C. J. (2001). The INK4a/ARF Network in Tumour Suppression. *Nature Reviews Molecular Cell Biology*.

[2] Read J., Wadt K. A., Hayward N. K. (2016). Melanoma Genetics. *Journal of Medical Genetics*.

[3] Primiero C. A., Maas E. J., Wallingford C. K., Soyer H. P., McInerney-Leo A. M. (2024). Genetic Testing for Familial Melanoma. *Italian Journal of Dermatology and Venereology*.

[4] Bishop D. T., Demenais F., Goldstein A. M. (2002). Geographical Variation in the Penetrance of CDKN2A Mutations for Melanoma. *Journal of the National Cancer Institute*.

[5] Cust A. E., Harland M., Makalic E. (2011). Melanoma Risk for CDKN2A Mutation Carriers Who Are Relatives of Population-Based Case Carriers in Australia and the UK. *Journal of Medical Genetics*.

[6] van der Rhee J. I., Krijnen P., Gruis N. A. (2011). Clinical and Histologic Characteristics of Malignant Melanoma in Families With a Germline Mutation in CDKN2A. *Journal of the American Academy of Dermatology*.

[7] Klatte D. C. F., Boekestijn B., Wasser M. N. J. M. (2022). Pancreatic Cancer Surveillance in Carriers of a Germline CDKN2A Pathogenic Variant: Yield and Outcomes of a 20-Year Prospective Follow-Up. *Journal of Clinical Oncology*.

[8] Sargen M. R., Helgadottir H., Yang X. R. (2022). Impact of Transcript (p16/p14ARF) Alteration on Cancer Risk in CDKN2A Germline Pathogenic Variant Carriers. *JNCI Cancer Spectrum*.

[9] Potjer T. P., Helgadottir H., Leenheer M. (2018). CM-score: A Validated Scoring System to Predict *CDKN2A* Germline Mutations in Melanoma Families From Northern Europe. *Journal of Medical Genetics*.

[10] Overbeek K. A., Rodríguez-Girondo M. D., Wagner A. (2021). Genotype-Phenotype Correlations for Pancreatic Cancer Risk in Dutch Melanoma Families With Pathogenic CDKN2A Variants. *Journal of Medical Genetics*.

[11] Gruis N. A., van der Velden P. A., Sandkuijl L. A. (1995). Homozygotes for CDKN2 (P16) Germline Mutation in Dutch Familial Melanoma Kindreds. *Nature Genetics*.

[12] Hille E. T., van Duijn E., Gruis N. A., Rosendaal F. R., Bergman W., Vandenbroucke J. P. (1998). Excess Cancer Mortality in Six Dutch Pedigrees With the Familial Atypical Multiple Mole-Melanoma Syndrome From 1830 to 1994. *Journal of Investigative Dermatology*.

[13] Chen S., Francioli L. C., Goodrich J. K. (2024). A Genomic Mutational Constraint Map Using Variation in 76,156 Human Genomes. *Nature*.

[14] Akbani R., Akdemir K., Aksoy B. (2015). Genomic Classification of Cutaneous Melanoma. *Cell*.

[15] Hayward N. K., Wilmott J. S., Waddell N. (2017). Whole-Genome Landscapes of Major Melanoma Subtypes. *Nature*.

[16] Reshkin S. J., Cardone R. A., Koltai T. (2024). Genetic Signature of Human Pancreatic Cancer and Personalized Targeting. *Cells*.

[17] Martínez-Jiménez F., Movasati A., Brunner S. R. (2023). Pan-Cancer Whole-Genome Comparison of Primary and Metastatic Solid Tumours. *Nature*.

[18] van den Akker P. C., Bolling M. C., Pasmooij A. M. G. (2022). Revertant Mosaicism in Genodermatoses: Natural Gene Therapy Right before Your Eyes. *Biomedicines*.

[19] Rothova M., Thompson H., Lickert H., Tucker A. S. (2012). Lineage Tracing of the Endoderm during Oral Development. *Developmental Dynamics*.

[20] Soukup V., Epperlein H. H., Horácek I., Cerny R. (2008). Dual Epithelial Origin of Vertebrate Oral Teeth. *Nature*.

[21] Tseng K. C., Crump J. G. (2023). Craniofacial Developmental Biology in the Single-Cell Era. *Development*.

[22] Martins F., Hiraki K. R., Mimura M. A. (2013). Heterotopic Gastrointestinal Mucosa in the Oral Cavity of Adults. *Oral Surgery, Oral Medicine, Oral Pathology and Oral Radiology*.

[23] Bains G. K., Pilkington R., Stafford J., Bhatia S. (2022). A Case Report of Oral Heterotopic Gastrointestinal Cysts (HGIC) and Review of the Literature. *Oral Surgery*.

[24] Jansen A. M., Crobach S., Geurts-Giele W. R. (2017). Distinct Patterns of Somatic Mosaicism in the APC Gene in Neoplasms From Patients With Unexplained Adenomatous Polyposis. *Gastroenterology*.

[25] Galandiuk S., Rodriguez–Justo M., Jeffery R. (2012). Field Cancerization in the Intestinal Epithelium of Patients With Crohn’s Ileocolitis. *Gastroenterology*.

[26] Bugter O., Spaander M. C. W., Bruno M. J., Baatenburg de Jong R. J., Amelink A., Robinson D. J. (2018). Optical Detection of Field Cancerization in the Buccal Mucosa of Patients With Esophageal Cancer. *Clinical and Translational Gastroenterology*.

